# Peripatric speciation in an endemic Macaronesian plant after recent divergence from a widespread relative

**DOI:** 10.1371/journal.pone.0178459

**Published:** 2017-06-02

**Authors:** Francisco J. Valtueña, Tomás Rodríguez-Riaño, Josefa López, Carlos Mayo, Ana Ortega-Olivencia

**Affiliations:** Área de Botánica, Facultad de Ciencias, Universidad de Extremadura, Badajoz, Spain; National Cheng Kung University, TAIWAN

## Abstract

The Macaronesian *Scrophularia lowei* is hypothesized to have arisen from the widespread *S*. *arguta* on the basis of several phylogenetic studies of the genus, but sampling has been limited. Although these two annual species are morphologically distinct, the origin of *S*. *lowei* is unclear because genetic studies focused on this Macaronesian species are lacking. We studied 5 *S*. *lowei* and 25 *S*. *arguta* populations to determine the relationship of both species and to infer the geographical origin of *S*. *lowei*. The timing of *S*. *lowei* divergence and differentiation was inferred by dating analysis of the ITS region. A phylogenetic analysis of two nuclear (ITS and ETS) and two chloroplast (*psbJ–petA* and *psbA–trnH*) DNA regions was performed to study the relationship between the two species, and genetic differentiation was analysed by AMOVA. Haplotype network construction and Bayesian phylogeographic analysis were conducted using chloroplast DNA regions and a spatial clustering analysis was carried out on a combined dataset of all studied regions. Our results indicate that both species constitute a well-supported clade that diverged in the Miocene and differentiated in the Late Miocene-Pleistocene. Although *S*. *lowei* constitutes a well-supported clade according to nDNA, cpDNA revealed a close relationship between *S*. *lowei* and western Canarian *S*. *arguta*, a finding supported by the spatial clustering analysis. Both species have strong population structure, with most genetic variability explained by inter-population differences. Our study therefore supports a recent peripatric speciation of *S*. *lowei*—a taxon that differs morphologically and genetically at the nDNA level from its closest relative, *S*. *arguta*, but not according to cpDNA, from the closest Macaronesian populations of that species. In addition, a recent dispersal of *S*. *arguta* to Madeira from Canary Islands or Selvagens Islands and a rapid morphological differentiation after the colonization to generate *S*. *lowei* is the most likely hypothesis to explain the origin of the last taxon.

## Introduction

Macaronesia is a floristic region comprising the north Atlantic archipelagos of Madeira, the Azores, the Selvagens, the Canary Islands and Cape Verde. These archipelagos, which are all of volcanic origin, are considered to be excellent models for studying plant evolutionary and dispersal processes because of their initial absence of life and high rate of endemism [1]. The origins of the Macaronesian islands span a wide range of ages, from 27 million years ago (Ma) for Grande Island in the Selvagens to 0.25 Ma for Pico in the Azorean archipelago [2, 3]. Plant colonisation has consequently been able to occur over extended periods of time, and, in the case of ancient colonisations, sufficient time has elapsed to allow different speciation processes to take place. The likelihood of island colonisation is thus dependent on distance to the continent, with colonisation of the islands closest to the mainland (Canary Islands) more feasible and predictable compared to the most distant archipelago (Azores) [4]. The existence of processes of speciation, by contrast, is largely determined by others factors, such as island age, size and diversity of habitats [5, 6].

Many studies have been published on the relationship of the various archipelagos to the mainland as well as relationships within and between archipelagos [7–10]. In the first case, the Canarian and Madeiran flora exhibits a clear affinity to the western Mediterranean, its main source region, whereas the Azores archipelago displays important relationships with Atlantic and Boreal Europe, and Cape Verde is related to the Saharan Tropical African flora [11–13]. Inter-archipelago dispersal events appear to be quite recent and have not resulted in any major radiation [8]; nevertheless, a large number of Macaronesian endemics are shared between two or more archipelagos, showing the importance of inter-archipelago dispersal to the Macaronesian biodiversity. The Canary Islands and Madeira share the largest number of endemic species (42), followed by Madeira and the Azores (7 endemics) [1]. Only one species, *Ranunculus cortusifolius*, is shared by these three archipelagos. In the case of *R*. *cortusifolius*, however, genetic differences among populations indicate that this taxon is actually a complex comprising several species having a common origin from a single colonisation of Macaronesia followed by subsequent inter-archipelago dispersal [14]. The close floristic relationship between the Canary Islands and Madeira shown in numerous studies is more often due to dispersal events from the Canaries to Madeira than vice versa [8–11, 15–20]. Studies focusing on plant taxa of the Azores have revealed their origins to be in south-western Europe [21], Madeira [22, 23] and the Canary Islands [19, 24].

In oceanic islands, the general pattern of formation of endemism usually involves one or a few colonisation events followed by evolutionary radiation, generally adaptive, into a range of new niches that usually imply a rapid rate of phenotypic evolution [6, 25]. Several phylogenetically related Macaronesian endemic species constituting monophyletic groups have been formed in this way (e.g. *Aeonium*, [26]; *Argyranthemum* [27]; *Echium* [16]; *Sonchus* [28]). In other cases, the formation of endemism appears to have involved other evolutionary processes (e.g. anagenesis) that result in the evolution of a single endemic taxon from a colonizing taxon ([6, 29]; for a discussion on the inconsistent use of the terms anagenesis and cladogenesis, see Vaux et al. [30] and reply by Allmon [31]); these processes are more prevalent on islands with low habitat heterogeneity and do not lead to an increased number of species [32]. Stuessy et al. [32] estimated the level of anagenetic speciation on several oceanic and continental archipelagos, taking into account three from Macaronesia (the Canary Islands, Madeira and Cape Verde); of these archipelagos, Madeira, which has the smallest number of islands and the lowest surface area and habitat heterogeneity, had the highest percentage of endemics (48%). Finally, from a biogeographic point of view, new species can originate in outlying or peripheral populations of the range of a species when gene flow to and from the main population is interrupted (peripatric or quantum speciation); this biogeographic model usually generates paraphyletic groups [33]. This isolation can be due to several factors, such as the ocean, which acts as a barrier to dispersion and gene flow between an island and the mainland and between islands.

The genus *Scrophularia* includes approximately 270 species [34] of primarily Holarctic distribution. In Macaronesia, 8 of 11 native species and subspecies are endemic. Seven of the endemic taxa (*Scrophularia hirta* and *S*. *racemosa* restricted to Madeira and *S*. *calliantha*, *S*. *glabrata* and three subspecies of *S*. *smithii* endemic to the Canary Islands) originated from a common ancestor after a single colonization event [35, 36], thus constituting another example of evolutionary radiation into Macaronesia. Dalgaard [37] described an eighth endemic species, the Madeiran *S*. *lowei*, which he associated with *S*. *arguta* that is widespread in Macaronesia, Africa from North Africa to the Horn of Africa, and the Arabian Peninsula and some isolated populations on the Iberian Peninsula [38].

Dalgaard [37] found that *Scrophularia lowei* and *S*. *arguta* can artificially hybridize, but their hybrids were always sterile. In a phylogenetic study, Scheunert and Heubl [36] supported that *S*. *lowei* is sister to *S*. *arguta*, while Navarro-Pérez et al. [35] dated the divergence of these two species to the Pliocene–mid Pleistocene (0.43–5.5 Ma) and hypothesized that peripatric speciation may explain the origin of the Madeiran *S*. *lowei* from its ancestor *S*. *arguta*. However, the number of individuals studied in either investigation was very limited (three or one individuals of *S*. *arguta*, respectively, and one individual of *S*. *lowei*). *Scrophularia arguta* has recently been shown to have distinct lineages in the Canary Islands, the Iberian Peninsula and north-western Africa [39], with their estimated divergence in the Pliocene (1.38–5.43 Ma). The relationship between the two species previously inferred using a low number of *S*. *arguta* individuals may thus have been distorted by the limited sampling. In addition, *S*. *lowei* was previously thought to be restricted to Madeira; however, a population from the Azorean São Miguel Island was discovered during our preliminary studies, thus extending the known geographical distribution of this species. Given this background, our main objectives were to: (1) determine the phylogenetic origin of *S*. *lowei* in regard to two exclusive hypotheses, namely, does *S*. *lowei* constitute a sister clade to *S*. *arguta*, or, instead, has it arisen via peripatric speciation from a lineage of *S*. *arguta*?, (2) analyse whether genetic differentiation between the two species mirrors their morphological differentiation, and (3) establish the geographical origin of *S*. *lowei*, taking into account the territories of the Canary Islands, north-western Africa or the Iberian Peninsula.

## Material and methods

### Species studied

**Fig 1:**
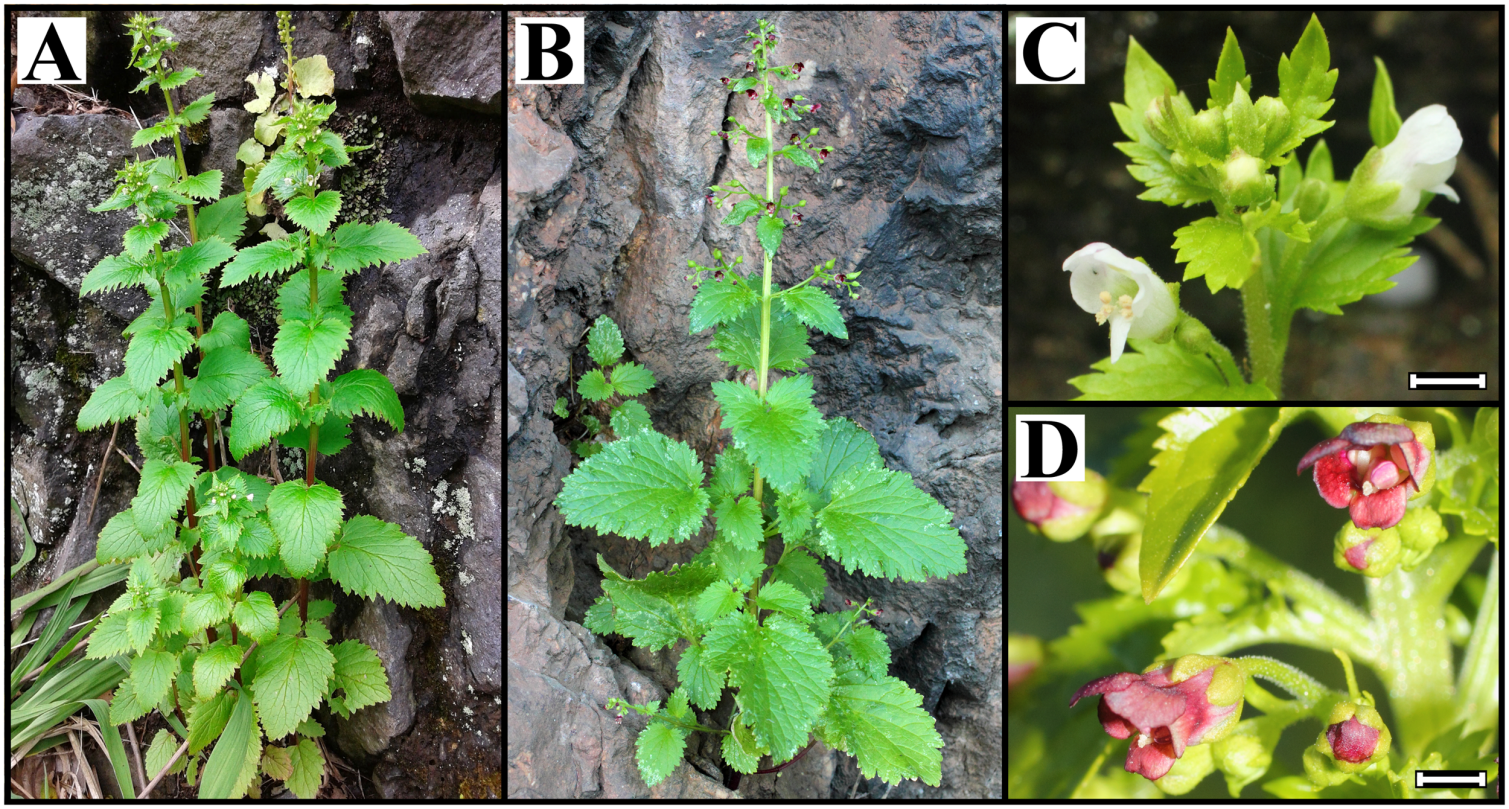
Habit and flower detail of *Scrophularia lowei* (A, C) and *S*. *arguta* (B, D). Scale bar = 2.5 mm.

*Scrophularia lowei* and *S*. *arguta* (Fig 1), annual species with the same chromosome number (2*n* = 40), are characterized by small flowers (corolla 3–5 mm), calyx-lobes possessing a keel, an androecium with a well-developed staminode and white pollen grains, and conical and beaked capsules [37]. The main morphological differences between these species are the presence or absence of cleistogamous flowers in shoots or in inflorescences arising from the plant base (present in *S*. *arguta* but not in *S*. *lowei*) and different corolla colours (white, with sometimes purplish striae and margins in *S*. *lowei vs*. brownish red in *S*. *arguta*; Fig 1). Other differences are pedicel length (shorter in *S*. *arguta*), bracteole size (larger in *S*. *lowei*) and density of glands on the stems (usually densely pubescent-glandular in *S*. *arguta vs*. sparsely glandular in *S*. *lowei*).

While *S*. *lowei* is a rare endemic of Macaronesia, with only a few populations known from the Madeiran archipelago [37, 40] and just one from the Azores (São Miguel Island, this study), *S*. *arguta* is widespread. In particular, *S*. *arguta* is present in Macaronesia (Selvagens and Canary islands and Cape Verde) and also ranges from north-western Africa to the Arabian Peninsula and the Horn of Africa, with some isolated populations on the Iberian Peninsula [38], although in all cases with low number of individuals per population. *Scrophularia lowei* inhabits rocky slopes, walls, cliffs, wastelands and banks, where it blooms from March to May. *Scrophularia arguta* has a similar ecological habitat in Macaronesia, but prefers basaltic cliffs in lowland xerophytic areas, especially *Euphorbia* communities on coastal rocky outcrops; in the Canary Islands, it blooms from February to May [37, 40, pers. observ.].

### Sampling strategy

**Fig 2:**
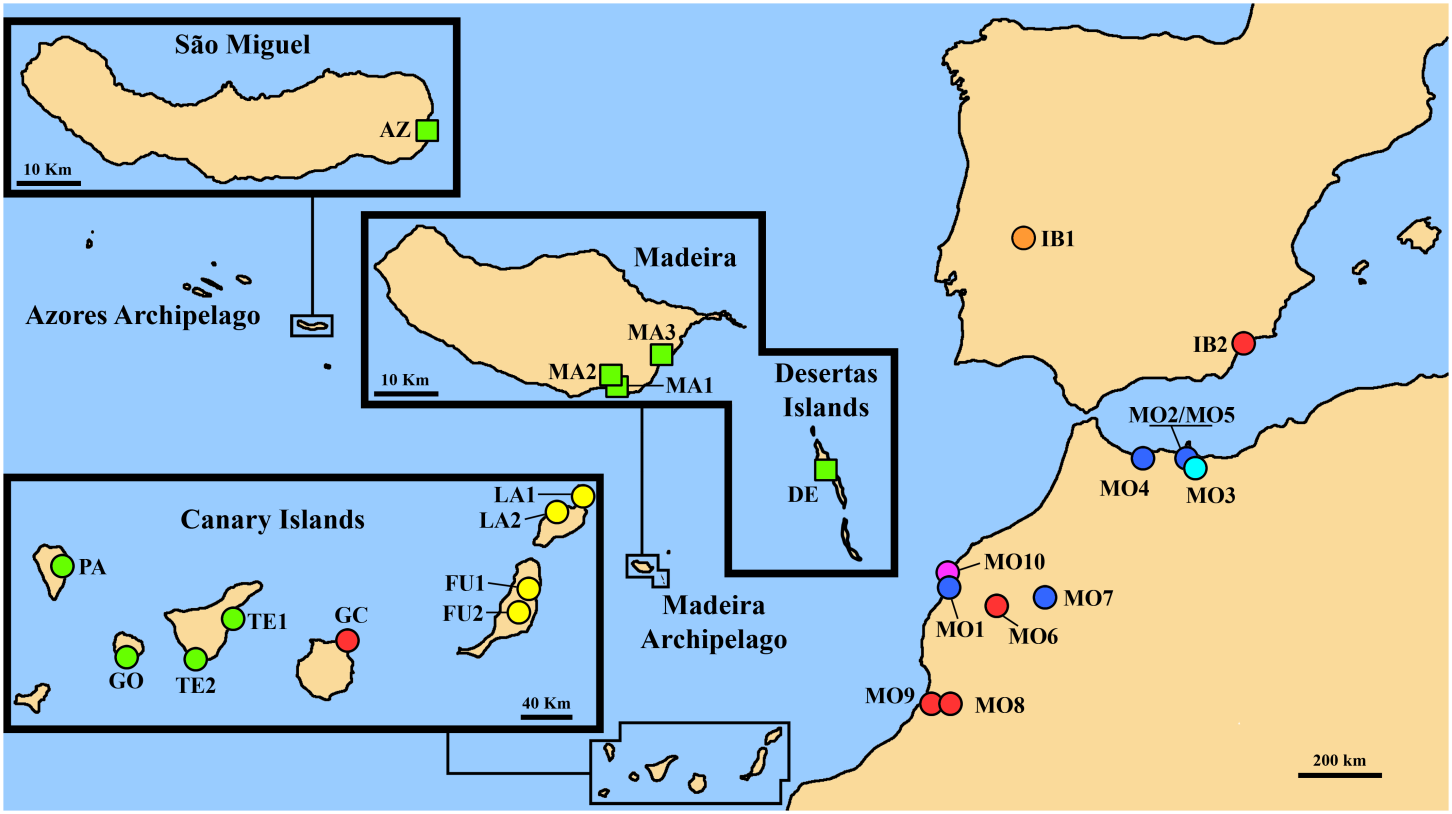
Location of studied populations (coded as in Table 1) of *Scrophularia lowei* (squares) and *S*. *arguta* (circles). Only populations of *S*. *arguta* from its western distribution range and Macaronesia are shown. Population colours indicating the main haplotype as in Fig 5A.

We sampled 26 individuals of *S*. *lowei* from five populations (three from Madeira and one from Deserta Grande in the Madeiran archipelago and one from São Miguel in the Azores) (Table 1; Fig 2). We also included 46 individuals from 25 populations of *S*. *arguta* sampled in a previous study [39]. Most of the *S*. *arguta* populations were located in the species’ westernmost distribution range (the areas closest to Madeira and Azores archipelagos), including nine populations from the Canary Islands (i.e. two populations each from Lanzarote, Fuerteventura and Tenerife, and one population each from Gran Canaria, La Gomera and La Palma), ten from Morocco and two from the Iberian Peninsula (Table 1, Fig 2). In each population, young leaves were collected and kept on silica gel until analysis. The number of studied individuals per population varied from one (in populations DE, GO, SA1, SA2 and SO where material from only one individual could be obtained/collected) to seven.

**Table 1:** Studied populations of *Scrophularia lowei* and *S*. *arguta*, including number of individuals studied (N) and haplotype groups (Hap).

Code	Location	Coordinates	Voucher	Collector*	N	Hap
*Scrophularia lowei*						
AZ	Azores Archipelago: São Miguel, Lombo Gordo	37°46.70’ N, 25°08.84’ W	UNEX 36159	FJV & CGR	7	G_1_
DE	Madeira Archipelago: Deserta Grande	32°30.68’ N, 16°30.22’ W	UNEX 35996	CA, SC, RJ, LM, MS & MV	1	G_1_
MA1	Madeira Archipelago: Madeira, Caniço Baixo	32°38.65’ N, 16°51.61’ W	UNEX 36010	JL & TRR	7	G_1_
MA2	Madeira Archipelago: Madeira, São Gonçalo	32°39.48’ N, 16°52.27’ W	UNEX 36004	JL & TRR	4	G_1_
MA3	Madeira Archipelago: Madeira, Santa Cruz	32°41.37’ N, 16°47.52’ W	UNEX 36003	JL & TRR	7	G_1_
*Scrophularia arguta*						
FU1	Canary Islands: Fuerteventura, Tetir	28°31.41’ N, 13°56.45’ W	UNEX 36128	JL & TRR	2	D_1_
FU2	Canary Islands: Fuerteventura, Tiscamanita	28°21.18’ N, 14°02.29’ W	UNEX 36129	JL & TRR	2	D_1_
GO	Canary Islands: La Gomera, Barranco de Guarimiar	28°04.45’ N, 17°13.81’ W	UNEX 36193	FJV & CM	1	G_1_
GC	Canary Islands: Gran Canaria, La Isleta	28°10.17’ N, 15°25.28’ W	UNEX 36192	FJV & CM	2	A_2_
IB1	Iberian Peninsula: Cáceres, Santiago de Alcántara	39°35.23’ N, 7°12.89’ W	UNEX 36131	AOO & FJV	2	C
IB2	Iberian Peninsula: Almería, Pulpí	37°26.38’ N, 1°44.30’ W	UNEX 36132	AOO & FJV	2	A_6_
LA1	Canary Islands: Lanzarote, Jameos del Agua	29°09.38’ N, 13°25.87’ W	UNEX 36135	JL & TRR	2	D_2_
LA2	Canary Islands: Lanzarote, Tinajo	29°03.61’ N, 13°41.46’ W	UNEX 36138	JL & TRR	2	D_1_
MO1	Morocco: Safi Cape	32°19.32’ N, 9°15.54’ W	UNEX 36084	AOO & FJV	2	E_1_
MO2	Morocco: Zegangane	35°09.80’ N, 3°00.68’ W	UNEX 36140	TRR, JL & FB	2	E_3_
MO3	Morocco: Hassi-Berkane	34°50.20’ N, 2°51.99’ W	UNEX 36141	TRR, JL & FB	2	F
MO4	Morocco: Had-Rouadi	35°08.15’ N, 4°09.40’ W	UNEX 36142	TRR, JL & FB	2	E_3_
MO5	Morocco: Beni-Sidel	35°11.48’ N, 3°03.02’ W	UNEX 36143	TRR, JL & FB	2	E_3_
MO6	Morocco: Sidi-Bou-Othmane	31°53.30’ N, 7°56.90’ W	UNEX 36144	AOO & FJV	2	A_5_
MO7	Morocco: Oued El-Abid Gorges	32°03.66’ N, 6°40.72’ W	UNEX 36145	AOO & FJV	2	E_2_ / E_1_
MO8	Morocco: Ouzaghar	29°44.77’ N, 9°05.90’ W	UNEX 36146	AOO & FJV	2	A_2_
MO9	Morocco: Oued Assaka	29°41.45’ N, 9°31.84’ W	UNEX 36147	AOO & FJV	2	A_2_
MO10	Morocco: Beddouza	32°32.88’ N, 9°16.34’ W	UNEX 36148	AOO & FJV	2	B
PA	Canary Islands: La Palma, Santa Cruz	28°42.31’ N, 17°45.46’ W	UNEX 36194	FJV & CM	7	G_1_ / G_2_
SA1	Saudi Arabia: Jabal Hada	21°16.68’ N, 40°22.58’ E	KSU 212279	AAG	1	A_4_
SA2	Saudi Arabia: Al-Baha	20°00.69’ N, 41°27.11’ E	KSU 17570	AHA	1	A_4_
SU	Sudan: Arkawit, Jebel Elsit	18°47.99’ N, 37°00.98’ E	UNEX 36150	UB, SAC & PK	2	A_3_
TE1	Canary Islands: Tenerife, Güimar	28°18.54’ N, 16°22.10’ W	UNEX 36151	JL & TRR	7	G_1_
TE2	Canary Islands: Tenerife, Pal-Mar	28°00.95’ N, 16°41.49’ W	UNEX 36152	JL & TRR	7	G_1_
SO	Yemen: Socotra, Fiheri Park	12°31.99’ N, 53°58.64’ E	UNEX 36153	JJA	1	A_1_

*AAG: A. Al-Ghuraibi; AHA: A.H. Alfarhan; AOO: A. Ortega-Olivencia; CA: C. Aedo; CGR: C.G. Relinque; FB: F. Bueno; CM: C. Mayo; FJV: F.J. Valtueña; JJA: J.J. Aldalsoro; JL: J. López; LM: L. Medina; MS: M. Sequeira; MV: M. Velayos; PK: P. Konig; RJ: R. Jardim; SAC: S.A. Chaudhary; SC: S. Castroviejo; TRR: T. Rodríguez-Riaño; UB: U. Bairele

### DNA extraction and sequencing

Genomic DNA was extracted using a Qiagen DNeasy Plant Mini kit (Qiagen GmbH, Hilden, Germany) following the manufacturer’s protocol. Two nuclear DNA (nDNA) and two chloroplast DNA (cpDNA) regions were amplified and sequenced using the following primers: ITS5 and ITS4 [41] for the nuclear internal transcribed spacer (ITS) region, 18S-2L [42] and ETS-Lar [43] for the nuclear external transcribed spacer (ETS) region, *psbJ* and *petA* [44] for the chloroplast *psbJ–petA* spacer region, and *psbA*-F and *trnH*-R [45] for the chloroplast *psbA–trnH* spacer region. Amplification was carried out as described by Valtueña et al. [39].

Sequencing in both directions was carried out by the Service of Applied Techniques to Biosciences (Extremadura University, Badajoz, Spain). Sequences were manually checked and edited using Sequencher version 4.10 (GeneCodes, Ann Arbor, MI, USA) and then manually aligned with MacClade version 4.08 [46]. A total of 104 sequences were newly generated for the four studied markers from the 26 sampled *S*. *lowei* individuals (Table 1). For populations in which more than one individual was studied, all unique sequences for each marker (or two representative sequences if all sequences were identical) were submitted to GenBank (see S1 Table for accession numbers). For each of the G-haplotype populations of *S*. *arguta* with more than two sampled individuals (PA, TE1 and TE2), we generated five additional sequences per marker. Because these sequences were identical to those generated in our previous study, they were not uploaded to GenBank (see S1 Table).

### Phylogenetic and genetic differentiation analyses

To estimate divergence times of *S*. *lowei* and *S*. *arguta*, a Bayesian phylogenetic analysis was performed in BEAST version 1.8.1 [47]. The analysis was carried out using ITS sequences from 131 *Scrophularia* taxa (including 10 sequences from *S*. *lowei* and 46 from *S*. *arguta*), and sequences of three related taxa in the Scrophulariaceae (two from *Verbascum* and one from *Teedia*) were used as outgroups (S1 and S2 Tables). From each population in which more than two individuals were sampled, only two individuals were included in the analysis because all the individuals either shared the same sequence (populations TE1, TE2, AZ and MA1) or only two different sequences were found (PA and MA3). The only exception was population MA2, in which three different sequences were found and used in the analysis. In this analysis, we considered three calibration points obtained from a previously constructed phylogeny of the genus *Scrophularia* [48] that included minimum stem-age constraints for Lamiales families and tribes based on five fossils following Vargas et al. [49] and Fernández-Mazuecos and Vargas [50]. The three calibration points implemented as normally distributed priors were (1) the split between *Teedia* and *Verbascum* + *Scrophularia* (26.77 ± 4.27 Ma), (2) the split between *Verbascum* and *Scrophularia* (15.92 ± 3.29 Ma), and (3) the crown age of *Scrophularia* (10.20 ± 2.36 Ma). The most suitable nucleotide substitution model was estimated using jModeltest 2.1.3 [51]. The GTR+I+G model was selected with the gamma distribution modelled with four categories. Both *Verbascum* + *Scrophularia* and *Scrophularia* were defined as monophyletic. A relaxed uncorrelated log-normal clock was used and a birth–death tree prior was set. Other priors were set to default values. Two Markov chain Monte Carlo [52] analyses were initiated on a random starting tree and run for 20 million generations each with a sampling frequency of 1000 generations. Satisfactory effective sample size was reached after assessing convergence in TRACER version 1.6 [53] as described in the BEAST manual [54]. After discarding the first 10% of sampled generations as burn-in, the two resulting tree files were combined in LogCombiner 1.8.1 [47]. The maximum clade credibility tree was summarized in TreeAnnotator version 1.8.1 [47] with a posterior probability limit of 0.90.

To infer relationships and divergence times between *S*. *lowei* and *S*. *arguta*, Bayesian phylogenetic analyses were conducted on two concatenated datasets: one consisting of the two nuclear regions (ITS and ETS) and the other composed of the two plastid regions (*psbJ*–*petA*/*psbA*–*trnH*). An incongruence length difference (ILD) test [55] was previously applied to the two nuclear regions of the nDNA dataset. The results of this test, which was performed in PAUP version 4b.10 [56] with 100 replicates, 10 random addition sequences, tree-bisection-reconnection (TBR) branch swapping on best trees only, and using the MULTREES option, confirmed the suitability of analysing the two nuclear markers together (P > 0.05). The Bayesian analyses were performed in BEAST and included at least two individuals from each population of *S*. *arguta* and *S*. *lowei* (except for GO, SA1, SA2, SO and DE, where only one individual was collected per population), with one sequence of *S*. *megalantha* used as outgroup. Because more than two different ITS/ETS sequences were found in three *S*. *lowei* populations, all unique sequences (three for AZ and four for MA2 and MA3) were used in the analysis of the nDNA dataset. Two calibration points were used in these analyses: the divergence age (10.50 ± 1.60 Ma) and the crown age (3.51 ± 1.18 Ma) of the clade constituted by *S*. *lowei* and *S*. *arguta*. The substitution models selected using jModeltest 2.1.3 [51] were GTR+I+G with four categories for the nDNA dataset and HKY+I for the cpDNA dataset, and a coalescent constant size tree prior was used. All other settings and calculation procedures were the same as in the previous dating analysis. The characteristics of the analysed datasets are detailed in S3 Table.

An ILD test with the same parameters used for the nDNA dataset was also used to test whether the two datasets (nDNA+cpDNA) could be combined. According to the ILD test (P > 0.05), the two datasets were congruent and were thus analysed together. The BI analysis was performed in BEAST using the same parameters used for analyses of individual datasets.

Relationships between *S*. *lowei* and *S*. *arguta* were additionally estimated by maximum likelihood (ML) analysis of the nDNA, cpDNA and combined datasets. The ML analyses were performed in RAxML version 8.1.11 [57] via XSEDE on the CIPRES Portal [58]. Automatic termination of bootstrapping was performed in RAxML, and GTR+G and GTR+I substitution models were selected for the nDNA and cpDNA datasets, respectively.

Genetic differentiation between *S*. *arguta* and *S*. *lowei* was assessed by analysis of molecular variation (AMOVA) as implemented in Arlequin 3.5.2.2 [59] using the cpDNA and nDNA datasets. AMOVA was run with 1000 permutations, and the significance of the coefficient *F*_ST_ was assessed with 100 permutations. For both datasets, two different approaches were run: (1) considering only species without taking populations into account, such that all sequences were assigned to either *S*. *arguta* or *S*. *lowei*, and (2) assigning populations to the two species (in which case, populations with only one sampled individual were removed from the dataset).

### Haplotype network, phylogeographic and spatial clustering analyses

Relationships among chloroplast haplotypes were analysed under statistical parsimony in TCS 1.21 [60] using a cpDNA matrix of 56 sequences (9 from *S*. *lowei*, 46 from *S*. *arguta* and 1 from the outgroup *S*. *megalantha*). Because the selected regions had several unambiguous complex indels (between 5 and 152 bp) as well as several polymorphic (polyA) regions that could not be coded unambiguously, two different approaches were used. In the first approach, a dataset consisting only of the unambiguous complex indels (S3 Table) coded as single characters was analysed with a connection limit of 12 steps. In the second approach, a dataset with all unambiguous mutations, including the complex indels coded as single characters, was analysed with a connection limit of 100 steps to include the outgroup. An analysis based on this second approach was additionally performed considering only *S*. *lowei* populations and those *S*. *arguta* populations having the same main haplotype (haplotype G; see Results), with the closest *S*. *arguta* population having a different haplotype used as an outgroup. For this last analysis, complex indels and polymorphic regions could be coded unambiguously. To include the outgroup, a connection limit of 50 steps was selected.

The dispersal and diffusion of *S*. *lowei* were analysed by Bayesian stochastic search variable selection (BSSVS [61]) of the discrete phylogeographic model as implemented in BEAST 1.8.1. This analysis was made on the cpDNA dataset and conducted using the same individuals studied in the haplotype network analysis. There were considered 2 geographic areas for *S*. *lowei* (Madeira and Azores archipelagos) and 11 geographic areas for *S*. *arguta* (the Iberian Peninsula, north-western Africa, eastern Africa, the Arabian Peninsula, Socotra Island and each Canarian island from which samples were collected). In the analyses, the coalescent model for the discrete geographical data used both symmetrical and asymmetrical substitution models, with all other settings and calculation procedures identical to those in the previous cpDNA analysis. Bayes factor (BF) analysis as implemented in SPREAD 1.0.7 [62] was used to identify well-supported geographical state transitions having strong posterior support (BF ≥ 3).

To determine the genetic structure of the combined region dataset, a spatial genetic mixture analysis [63] was performed using ‘Bayesian analysis of population structure’ (BAPS, version 6.0) [64] with the population coordinates incorporated into the analysis and 30 selected as the maximum number of populations present in the sample.

## Results

**Fig 3:**
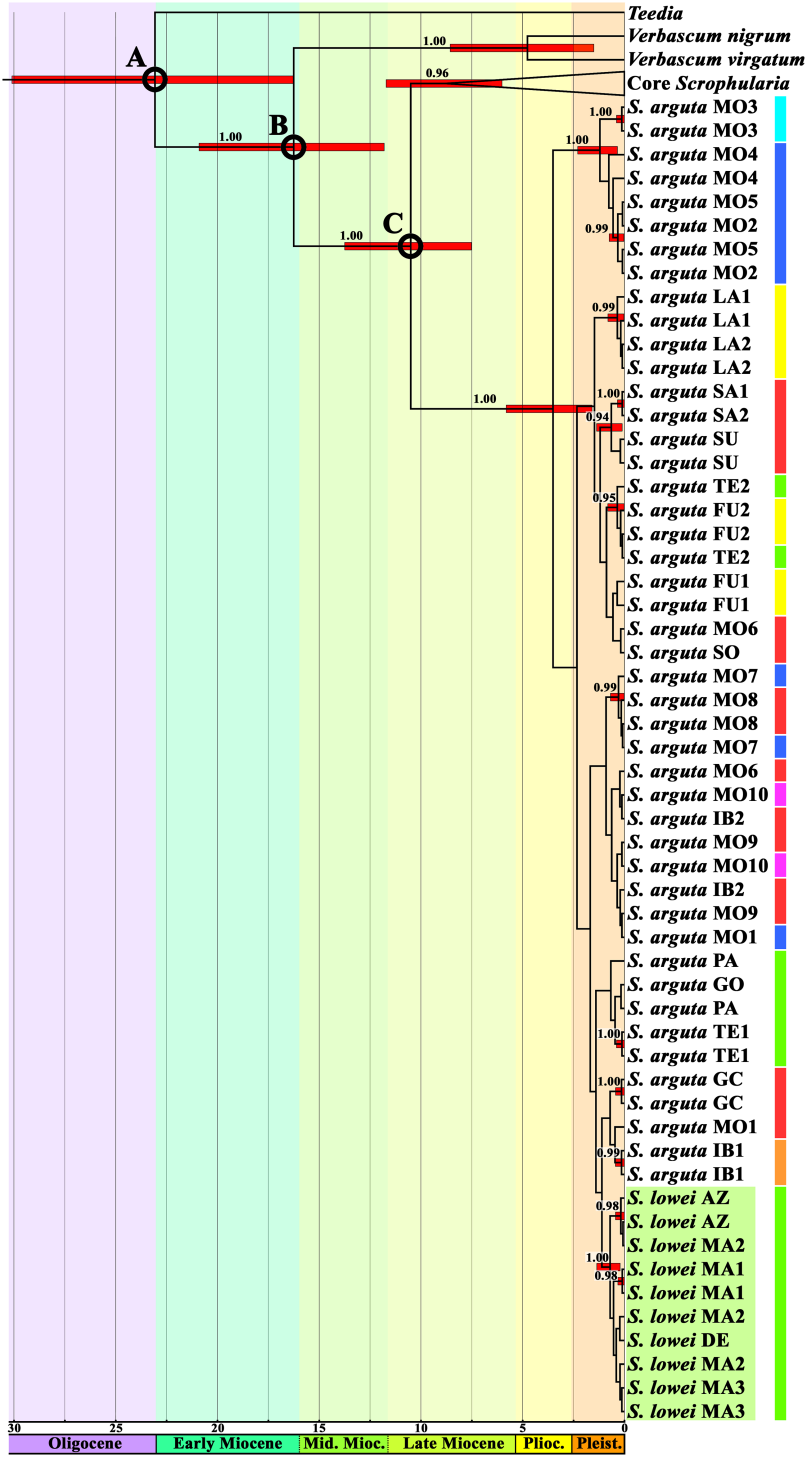
Molecular dating of *Scrophularia* based on ITS sequence variation using BEAST. Core *Scrophularia* includes all analysed *Scrophularia* species except *S*. *arguta* and *S*. *lowei*; the complete tree is shown in S1 Fig. Clade posterior probabilities ≥ 0.90 are indicated above branches. The 95% posterior density distribution of node ages is superimposed in red on branches with a PP ≥ 0.90. Calibration points used in the analyses are indicated (A, 26.77 ± 4.27 million years ago [Ma]; B, 15.92 ± 3.29 Ma; C, 10.20 ± 2.36 Ma). *Scrophularia lowei* sequences are indicated by the light green background. The scale is in millions of years. Abbreviations: Pleist., Pleistocene; Plioc., Pliocene; Mid. Mioc., Middle Miocene. Colours on the right correspond to the main haplotype indicated as in Fig 5A.

In the ITS-based Bayesian tree, *S*. *lowei* and *S*. *arguta* together constitute a highly supported clade (posterior probability, PP = 1.00) that is the most basal lineage within the genus *Scrophularia* (Fig 3, S1 Fig). The dating analysis placed the origin of this clade in the Miocene (10.50 Ma; 7.52–13.75 Ma, 95% highest posterior density confidence interval, HPD) and its diversification in the Late Miocene-Pleistocene (3.51 Ma, 1.59–5.81 Ma HPD). All *S*. *lowei* individuals constitute a highly supported clade (PP = 1.00), whose differentiation was dated to 0.70 Ma (0.21–1.36 Ma HPD). The tree is insufficiently resolved to allow determination of the relationship of the *S*. *lowei* clade to the various *S*. *arguta* clades and its time of divergence.

**Fig 4:**
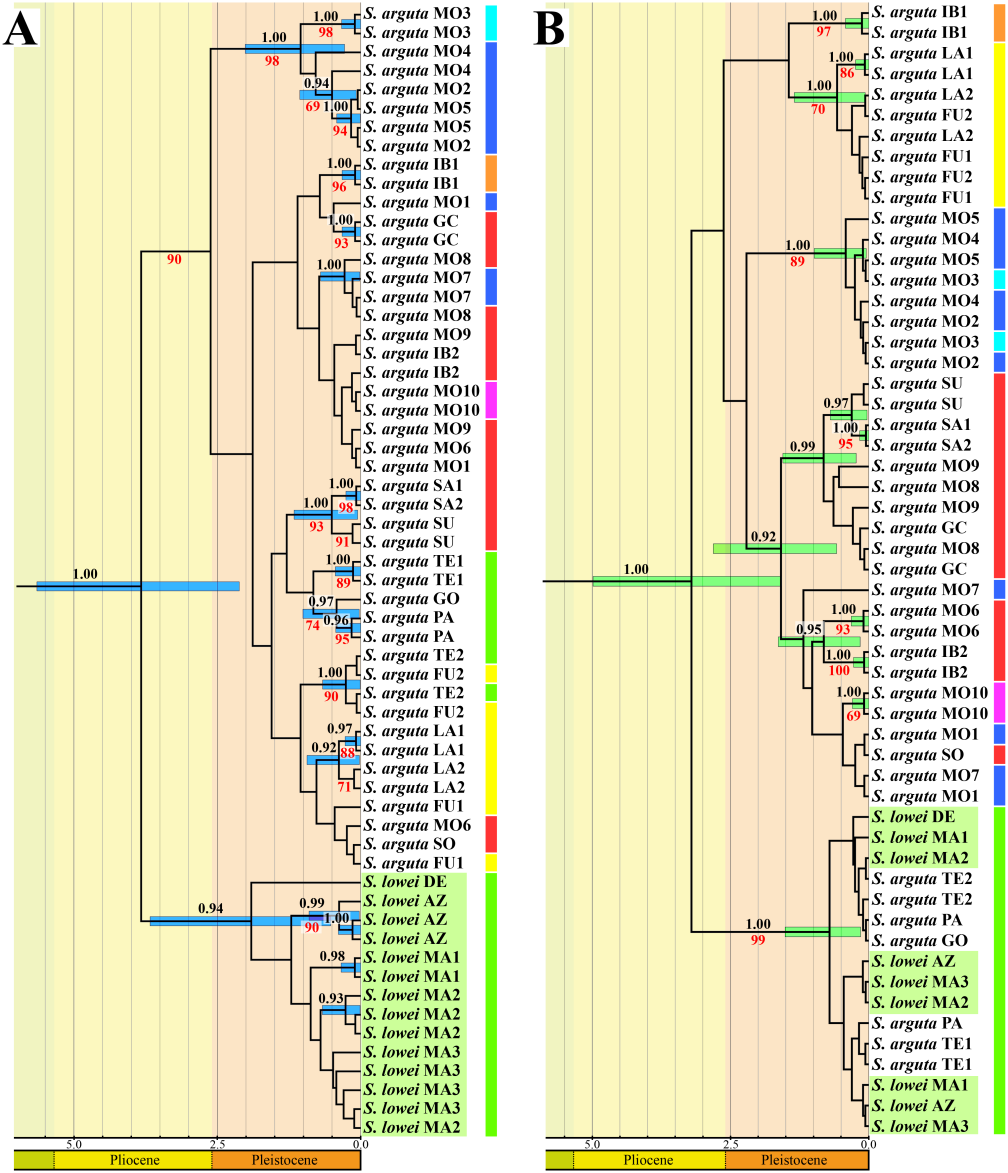
BEAST chronogram of *Scrophularia lowei* and *S*. *arguta* based on (A) nuclear (ITS/ETS) and (B) chloroplast (*psbA–trnH/psbJ–petA*) DNA sequences. Black and red numbers above and below branches are posterior probability (PP) and maximum likelihood (ML) bootstrap (BS) values, respectively. Only values corresponding to a PP ≥ 0.90 and a ML BS ≥ 65 are shown. The light green background indicates *S*. *lowei* populations. Colours on the right correspond to the main haplotype indicated as in Fig 5A.

Bayesian analyses of the individual nDNA and cpDNA datasets produced different results. In the nDNA tree (Fig 4A), all *S*. *lowei* individuals constitute a marginally supported clade (PP = 0.94) with a diversification age of 1.91 Ma (0.52–3.67 Ma HPD), but the poor resolution of the tree does not allow the relationship of this clade to the different *S*. *arguta* lineages to be inferred. Within the *S*. *lowei* clade, the Azorean individuals cluster together with strong support (PP = 0.99). In the cpDNA tree, all *S*. *lowei* individuals are included in a well-supported clade (PP = 1.00) along with individuals of *S*. *arguta* from the three most western sampled Canary Islands (Tenerife, La Gomera and La Palma), which pinpoints the differentiation of this clade to the Pleistocene (0.71 Ma, 0.15–1.51 Ma HPD) (Fig 4B). The lack of resolution in the tree prevents inferences regarding relationships among the different populations in this clade and between this clade and other *S*. *arguta* lineages (Fig 4B).

The tree generated by analysis of the combined dataset (nDNA+cpDNA), which is similar to the chloroplast tree (S2 Fig), includes a highly supported clade (PP = 1.00) consisting of all *S*. *lowei* individuals and the three most western sampled Canary Island *S*. *arguta* populations. This clade diversified in the Pleistocene (1.10 Ma, 0.32–2.05 Ma HPD). Within this clade, the *S*. *lowei* individuals are clustered together with high support (PP = 1.00; differentiation 0.54 Ma, 0.13–1.07 Ma HPD). As in the cpDNA tree, the time of the diversification of this clade as well as its relationship to the remaining clades, which comprise only *S*. *arguta* individuals, is unresolved.

The results of ML analyses were similar to those generated by the Bayesian analyses, but the trees were poorly resolved (Fig 4 and S2 Fig).

**Fig 5:**
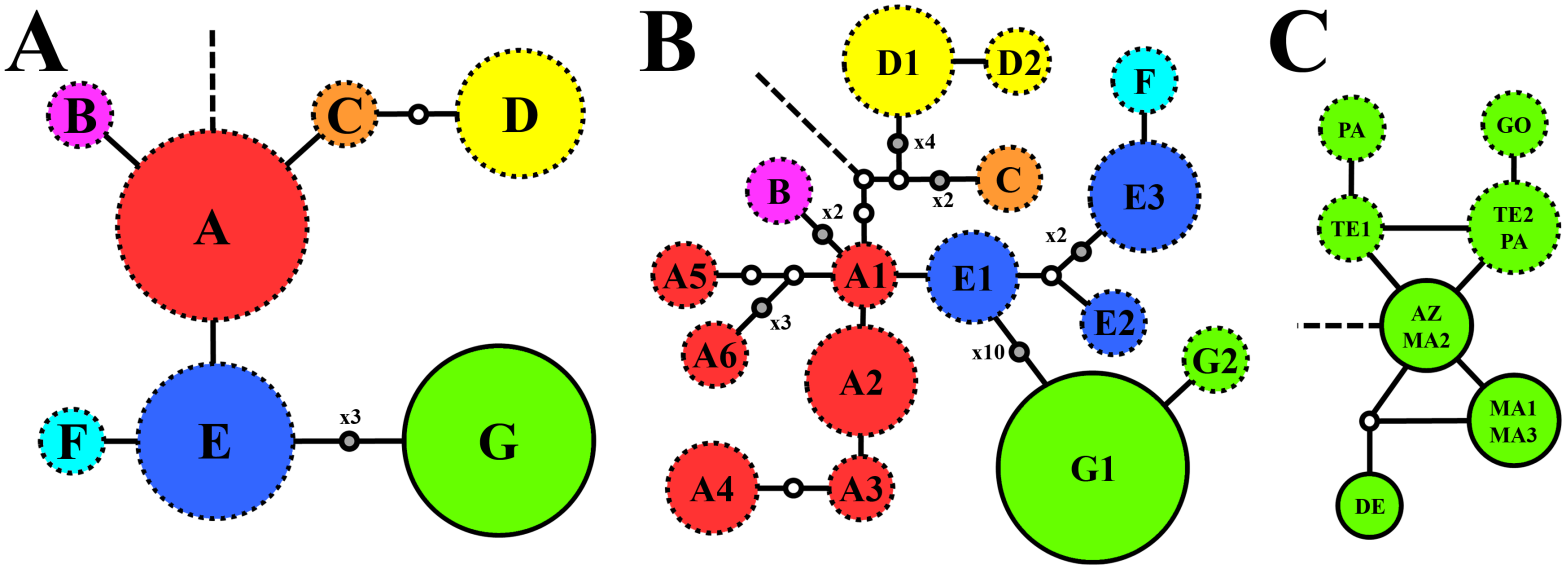
TCS statistical parsimony network of chloroplast DNA haplotypes found in *Scrophularia arguta* and *S*. *lowei* in the matrix considering only complex gaps as mutational steps (A) and the matrix including all unambiguous mutations (B, all haplotypes; C, only haplotype G). In these analyses, gaps were coded as single mutations. Dashed lines indicate connections to the outgroup (*S*. *megalantha* in A and B; haplotype E1 in C). Haplotypes found in *S*. *lowei* populations are indicated by solid margins. Small circles represent inferred mutational steps. The size of a given haplotype symbol indicates the relative number of populations harbouring that haplotype. Haplotype abbreviations (A, B) and population codes (C) are the same as in Table 1.

The close relationship revealed between *S*. *lowei* and populations of *S*. *arguta* from the western Canary Islands was corroborated by the results of the haplotype network analyses, where these populations were found to share haplotype G (Fig 5A, Table 1), the most divergent haplotype in the *S*. *lowei* + *S*. *arguta* group (Fig 5B). The analysis focused only on haplotype G distinguished seven subtypes based on variation in four different positions of the matrix (Fig 5C, S4 Table)—three in *S*. *lowei* and four in *S*. *arguta* populations—with no shared subtypes among populations of both species. The closest haplotype to the remaining *S*. *arguta* haplotypes was found in the Azores and Madeira (Fig 5C). In all populations with haplotype G, all analysed individuals in a given population had the same sequence. The one exception was in population PA, where one individual had a divergent sequence that was shared with another population from Tenerife.

When populations were ignored, AMOVA indicated that the genetic variability found in the *S*. *arguta*–*S*. *lowei* complex is mainly due to intraspecific differences (76.5% in cpDNA and 92.5% in nDNA), being much smaller the proportion of genetic variability explained by the species, which was higher in the cpDNA than in the nDNA (23.5% *vs*. 7.5%) (Table 2). When populations were considered, most variability was explained by differences at the inter-population level (Table 2). *F*_ST_ values were high in both analysed regions (cpDNA: 0.90465; nDNA: 0.66021), indicating the existence of a strong population structure (Table 2).

**Table 2:** Analysis of molecular variance (AMOVA) of nuclear DNA (nDNA) and chloroplast DNA (cpDNA) between *Scrophularia arguta* and *S*. *lowei*.

	Source of variation	d.f.	Sum of squares	Variance components	% of variation	*F*_ST_	P
Only species considered							
cpDNA	Among species	1	5.015	0.12628	23.50	0.2350	0.000
	Within species	85	34.950	0.41118	76.50		
nDNA	Among species	1	1.838	0.03768	7.50	0.07505	0.000
	Within species	85	39.472	0.46438	92.50		
Population considered into species							
cpDNA	Among species	1	5.185	0.08339	15.86	0.90465	0.000
	Among populations within species	23	29.250	0.39221	74.60		
	Within populations	57	2.857	0.05013	9.53		
nDNA	Among species	1	1.936	–0.00266	–0.54	0.66021	0.000
	Among populations within species	23	27.222	0.32649	66.56		
	Within populations	57	9.500	0.16667	33.98		

Phylogeographic reconstruction based on cpDNA identified north-western Africa as the ancestral region of the clade constituted by *S*. *lowei* + western Canarian *S*. *arguta* populations, with a PP of 0.49 under the symmetrical model and 0.61 under the asymmetrical one (S3 Fig). In both analyses, *S*. *lowei* populations from the Azores and Madeira were inferred to have been derived via two separate dispersal events from Canarian populations. Under both models, the dispersal to Madeira was suggested to have occurred from Tenerife (S3 Fig). The ancestral location of the Azores population was inferred to be Tenerife under the symmetrical model (Figure A in S3 Fig), and La Palma under the asymmetrical model (Figure B in S3 Fig). The symmetrical model detected six significant connections (dispersal routes) among geographical regions associated with *S*. *lowei* populations *vs*. nine connections identified using the asymmetrical model (S5 Table).

The best partition of the spatial clustering analysis yielded two clusters (log(marginal likelihood) = –2797.8527) that did not correspond to the two species. Populations of *S*. *lowei* grouped in one cluster with the western Canarian populations of *S*. *arguta*, whereas the remaining *S*. *arguta* populations constituted the other cluster. Remarkably, assignment of population DE of *S*. *lowei* to the group comprising only *S*. *arguta* lowered more the likelihood to a greater extent than changes in the position of any western Canarian populations of *S*. *arguta* (S6 Table).

## Discussion

### *Scrophularia lowei*–*S*. *arguta* relationships

In this study, we have shown that *S*. *lowei* is differentiated genetically from *S*. *arguta*, except at chloroplast loci. *Scrophularia lowei is* a species with high phenotypic and geographic differentiation with respect to *S*. *arguta*. Their phenotypic differentiation is supported by clear morphological differences, while their geographical differentiation is reflected in their distribution in different archipelagos: *S*. *lowei* is restricted to the Azores and Madeira, whereas *S*. *arguta* is found in the other Macaronesian archipelagos. According to phylogenetic analyses, these two taxa constitute a well-supported clade and are not independent sister lineages; consequently, they should not be recognised as autonomous species if monophyly is considered an essential requirement for species circumscription. In the literature, however, examples of plant and animal taxa are emerging that are not monophyletic because of speciation processes that do not involve cladogenesis or bifurcating trees (e.g. [65, 66]).

In addition to the morphological differences pointed out by Dalgaard [37], the two species differ with respect to several reproductive characteristics. In particular, *S*. *arguta* is an amphicarpic plant, having mainly chasmogamous (but sometimes cleistogamous) aerial flowers and cleistogamous basal and/or underground flowers. By contrast, *S*. *lowei* lacks basal or subterranean cleistogamous flowers, but sometimes with cleistogamous flowers produced only in ordinary cymes. As a consequence, *S*. *arguta* has dimorphic flowers (whitish, cleistogamous flowers lacking staminodes *vs*. brownish-red chasmogamous flowers with staminodes) and dimorphic fruits, with the cleistogamous fruits being thinner and with a lower number of seeds than the chasmogamous ones [38]. Both selfing species produce abundant fruits by spontaneous self-pollination; the typical protogyny of the genus is not effective because styles never become deflexed and the length of the stigmata places this structure at the same level as the open anthers [37].

The *S*. *arguta–S*. *lowei* group is reproductively isolated from all other Macaronesian *Scrophularia* species as a result of cross-incompatibility [37]. Although artificial hybrids within this group can be easily obtained, a distinct barrier to gene exchange exists between the two species that prevents the growth of F_1_ hybrid plants, which either die before flowering or shortly thereafter and are consequently completely sterile.

Our results provide evidence for a close relationship between the two species, as they constitute a well-supported clade. Molecular dating using the ITS region estimated an origin in the Late Miocene-Pleistocene, with all *S*. *lowei* individuals grouped in a well-supported clade (PP = 1.00) that differentiated during the Pleistocene (0.70 Ma, 0.21–1.36 Ma HPD). Because of a lack of resolution at the base of the clade, however, the relationship between *S*. *lowei* and the different *S*. *arguta* lineages could not be determined by this analysis. By contrast, analysis of chloroplast regions (maternally inherited) yielded a better-resolved tree, with *S*. *lowei* individuals grouped in a well-supported clade (PP = 1.00; ML bootstrap = 99%) with *S*. *arguta* individuals from the western Canarian islands (Tenerife, La Gomera and La Palma). Because genetic structure detected using paternally or biparentally inherited markers is considerably weaker than that based on maternally inherited markers [67], the latter type of markers is more suitable to infer historical evolution and relationships among populations [68].

Topological incongruence, frequently observed among trees derived using different genomic regions, is generally interpreted to be a consequence of hybridization, introgression or incomplete lineage sorting [69–74]. In our study, the absence of differentiation in chloroplast sequences among *S*. *lowei* and western Canarian populations of *S*. *arguta* may indicate recent hybridization between populations from these regions. As indicated above, however, hybrid offspring of the two species are sterile [37], which eliminates hybridization as an explanation for the observed differences in the two markers. In addition, the strong population structure (i.e. limited inter-population gene flow) detected and the fact that all individuals from a given population had the same cpDNA sequence—excluding one of the seven studied individuals in population PA—do not support the hypothesis of a hybrid origin for the populations with haplotype G.

An alternative hypothesis is that both population groups have diverged so recently that insufficient time has elapsed for differentiation of cpDNA regions. The close relationship of *S*. *lowei* to some Canarian populations of *S*. *arguta* would thus be consequence of incomplete lineage sorting, a phenomenon in which a studied gene or DNA region has diverged before species differentiation. In other words, the region is polymorphic in the ancestral population or species and after speciation the same gene pool is shared by the new species or populations [75]. In our case, the great differentiation among haplotypes in *S*. *arguta* is consistent with a haplotype differentiation predating the speciation process that generated *S*. *lowei*. This idea is also supported by the spatial clustering analysis, in which both groups of populations were identified as belonging to the same genetic group.

The large differentiation in nDNA regions might be a possible consequence of these two species’ annual and selfing habits [37]. Mutations arising in the nuclear genome would thus have become rapidly fixed in a given population, thereby leading to differentiation of nDNA regions [76]. This idea is supported by the high level of inter-population differentiation uncovered by the AMOVA and the high detected *F*_ST_ values. In addition, the absence of a relationship between *S*. *lowei* and western Canarian *S*. *arguta* populations at the nDNA level might be due to the existence of gene flow between the two Canarian clades; this influence may be reflected in the nuclear tree, where one population from Tenerife and one from Fuerteventura constitute a well-supported clade.

In phylogenetic analyses, the ITS region presents additional difficulties, such as the amplification of homologous genes or pseudogenes [77, 78]. With respect to our results, this possibility can be eliminated from consideration for two reasons. First, we never observed double bands on electrophoretic gels following amplification. Second, we sequenced all samples in both directions, but found no polymorphism in the sequences suggesting homologous gene amplification. Because the nuclear genome is recombinant and fixation of neutral mutations within populations is favoured by the reproductive traits of both species, mutation rates in the studied regions could have been overestimated; consequently, the obtained differentiation age of *S*. *lowei* should be taken with caution.

Clear morphological differentiation without genetic divergence, a phenomenon also observed in other studies, has been interpreted as a very early stage of differentiation [79–81]. Considering the low level of cpDNA differentiation between *S*. *lowei* and western Canarian populations of *S*. *arguta*, we conclude that these two groups of populations have recently diverged and that the absence of genetic differentiation at that level is only due to the factor of time [82]. While marked genetic differentiation has not been accompanied by morphological differentiation (morphological stasis [39]) within the bulk of the *S*. *arguta* complex, the opposite pattern is interestingly observed in the north-western region of its distributional range, where morphological differentiation has progressed much more rapidly than genetic differentiation. Natural hybridization within this group of “species” is virtually impossible because they inhabit different islands, similar to the situation observed in some *Bidens* species in the Hawaiian Islands [83].

The range of both taxa and the close phylogenetic affinity of *S*. *lowei* to the western Canarian *S*. *arguta* populations imply that *S*. *lowei* originated through peripatric speciation from a lineage of the latter group of populations. This finding supports the hypothesis of Navarro-Pérez et al. [35], who only studied one individual of each species. Studies have indicated that the usual initial result of peripatric speciation is that the widespread species becomes paraphyletic (as in *S*. *arguta* in our study), with monophyly being achieved only after enough time has elapsed for lineage sorting and extinction to take place [33].

Our results imply that *S*. *arguta* is currently a paraphyletic taxon that needs to encompass *S*. *lowei* to be considered monophyletic. However, the existence of different clades with clear geographical circumscription into *S*. *arguta* may imply that this species is actually a complex of different genetic groups, a possibility that was recently suggested by a study of the colonization of the Canary Islands by this species [39].

### Geographical origin of *Scrophularia lowei*: Macaronesian colonization

*Scrophularia lowei* has been considered to be endemic to the Madeiran archipelago. In this study, however, its distribution range has been slightly expanded, with a single population (see below) having been located on São Miguel island (Azores archipelago). Chloroplast markers indicate a close relationship between *S*. *lowei* and western Canarian *S*. *arguta* populations involving a recent divergence among the populations with haplotype G. An affinity between Canary Islands and Madeira has been previously indicated for the genus *Scrophularia* [35, 36]. Unfortunately, the poor resolution obtained in those studies hindered determination of the dispersal route between archipelagos and identification of the archipelago initially colonized from the mainland. Although our results similarly do not allow the geographical origin of *S*. *lowei* or the direction of dispersal to be determined with certainty, we propose two main hypotheses based on our data.

The first hypothesis involves initial colonisation of the Canary Islands by an ancestor with haplotype G from the mainland (or, alternatively, colonisation of the Selvagens, located between Madeira and the Canary Islands). A subsequent, recent dispersal from these islands (Canaries or Selvagens) to Madeira was followed by extensive morphological differentiation as the introduced population rapidly adapted to the different evolutionary pressures existing on Madeira. This hypothesis, which best fits the observed morphological pattern of differentiation, is supported by multiple studies inferring the dispersal of various taxa from the Canary Islands to Madeira [8–10, 19, 20].

Our second hypothesis entails initial colonisation of Madeira (or the Azores) from the mainland (north-western Africa), with the Canarian populations then originating after dispersal from Madeira. This hypothesis is supported by the results of our BSSVS analysis using the asymmetrical model, which implies that the location of the ancestor of the clade constituted by *S*. *lowei* and western Canarian *S*. *arguta* was Madeira, and by the generated haplotype network, where the subtype of haplotype G connected to the outgroup is found in Madeira and the Azores. Other studies focused on Macaronesian genera such as *Tolpis* [22] and *Festuca* [84] have also considered the possibility of dispersal from Madeira to the Canary Islands. This second hypothesis, however, implies that morphological differentiation in Madeira/Azores giving rise to *S*. *lowei* took place after recent dispersal to the Canary Islands. Considering that the populations with haplotype G have long been isolated from the remaining populations of *S*. *arguta* (i.e. since divergence in the Pliocene [39]), this hypothesis implies that the Madeiran/Azorean populations have been in morphological stasis for an extended period of time.

Although dispersal events related to colonization of oceanic islands are generally unpredictable [85], events involving shorter dispersal distances are considered to be more likely [22]. In our case, our first hypothesis (north-western Africa → Canary Islands → Madeira) is more feasible because it involves a shorter distance than that of the second hypothesis (north-western Africa → Madeira → Canary Islands) (760 *vs*. 1070 km). The most plausible hypothesis is thus the one placing the origin of *S*. *lowei* in either the western Canary Islands or in the Selvagens Islands, from whence a relatively recent dispersal event northward to Madeira was followed by rapid morphological differentiation *in situ*. This hypothesis involves the shortest dispersal distance and best fits the process of *S*. *lowei* morphological differentiation.

Finally, the colonization of São Miguel Island (Azores archipelago), which has increased the range of *S*. *lowei*, is probably very recent. The only population found on this island comprises approximately 300 individuals inhabiting basaltic rocks along the margins of a road. Given this location, the population may be the result of an accidental human introduction. This speculation is supported by the fact that more than 80% of the Azorean flora is non-native (only 205 of 1110 known species are native [86]). If this population is native, however, other populations may exist in steep inaccessible areas of this or other Azorean islands. In this context, we note that the latest checklist of Azorean biodiversity reveals a roughly 15% increase in the number of vascular plant taxa [87].

## Conclusions

A close relationship exists between western Canarian *S*. *arguta* populations and *S*. *lowei*, with both having recently diverged from a common ancestor. This close relationship supports the hypothesis that *S*. *lowei* originated by peripatric speciation from a *S*. *arguta* lineage. The results obtained from the cpDNA data suggest that *S*. *arguta* without the inclusion of *S*. *lowei* is a paraphyletic species, consistent with the observation that paraphyly is a relatively common phenomenon following recent speciation. Finally, our study supports an affinity between the Canary Islands and Madeira, and the most likely hypothesis to explain the origin and evolution of *S*. *lowei* involves a first colonization of Madeira from islands located south of its current range (Canary Islands or Selvagens Islands) and a rapid morphological differentiation after the dispersal.

## Supporting information

S1 Table Studied materials of *Scrophularia lowei*, *S*. *arguta* and the outgroup taxon (*S*. *megalantha*). Studied materials of *Scrophularia lowei*, *S*. *arguta* and the outgroup taxon (*S*. *megalantha*), including population codes (as in Table 1), locations and GenBank accession numbers of analysed DNA sequences.
(PDF)

S2 Table Taxa included in the ITS dataset. Taxa included in the ITS dataset for dating the origin and diversification of *Scrophularia lowei*, including GenBank accession numbers (GBN). *Scrophularia lowei* and *S*. *arguta* samples used in this analysis were the same as those given in S1 Table.
(PDF)

S3 Table Characteristics of DNA sequence datasets. Characteristics of DNA sequence datasets and number of unambiguous indels used in the analysis of *Scrophularia lowei* and *S*. *arguta*.
(PDF)

S4 Table Chloroplast DNA sequence variation in populations with haplotype G. (PDF)

S5 Table Bayes factor (BF) support for significant connections (BF > 3) between geographical areas. Bayes factor (BF) support for significant connections (BF > 3) between geographical areas based on BSSVS analysis of chloroplast DNA in *Scrophularia lowei* and *S*. *arguta* using symmetrical and asymmetrical models. Only connections including *S*. *lowei* populations are indicated.
(PDF)

S6 Table Assignment of populations to cluster and changes in logarithm of marginal likelihood (log(ML)) when populations were transferred to the no selected cluster. (PDF)

S1 Fig BEAST chronogram of *Scrophularia* based on ITS sequence variation. BEAST chronogram of *Scrophularia* based on ITS sequence variation. Posterior probabilities of clades are indicated above branches (only PP ≥ 0.90). The 95% posterior density distribution of node ages is shown in the node bars (only branches with a PP ≥ 0.90). The scale is in million years. Arrows indicate calibration points used in the analysis (A, 26.77 ± 4.27 Ma; B, 15.92 ± 3.29 Ma; C, 10.20 ± 2.36 Ma).
(TIF)

S2 Fig BEAST chronogram of *Scrophularia lowei* and *S*. *arguta* based on the analysis of the combined cpDNA-nDNA dataset. Black and red numbers above and below branches are posterior probability (PP) and maximum likelihood (ML) bootstrap (BS) values, respectively. Only values corresponding to a PP ≥ 0.90 and a ML BS ≥ 65 are shown. The light green background indicates *S*. *lowei* populations. Colours on the right correspond to the main haplotype indicated as in Fig 5.
(TIF)

S3 Fig Maximum clade credibility tree generated by BSSVS analysis of cpDNA. Maximum clade credibility tree generated by BSSVS analysis of cpDNA in *Scrophularia lowei* and *S*. *arguta* considering symmetrical (A) and asymmetrical (B) models. Branches are colored according to highest probability inferred ancestral geographical range. Highest probability of geographical range is indicated above branches (only values < 1.00).
(TIF)
