# Supplementary material for: Peripatric speciation in an endemic Macaronesian plant after recent divergence from a widespread relative
Source: PLoS One. 2017 Jun 2;12(6):e0178459. doi: 10.1371/journal.pone.0178459 (PMC5456078; doi:10.1371/journal.pone.0178459)
Supplement: S1 Table — Studied materials of Scrophularia lowei, S. arguta and the outgroup taxon (S. megalantha), including population codes (as in Table 1), locations and GenBank accession numbers of analysed DNA sequences. (PDF) [file pone.0178459.s001.pdf]

S1 Table. Studied materials of *Scrophularia lowei, S. arguta* and the outgroup taxon (*S. megalantha*), including population codes (as in Table 1), locations and GenBank accession numbers of analysed DNA sequences.

Code	Location	ETS	ITS	*psbA–trnH*	*psbJ–petA*
		Scrophularia lowei			
AZ	Azores Archipelago: São Miguel, Lombo Gordo	**MF115719**	**MF115733**	**MF115743**	**MF115752**
		**MF115720**	**MF115734**	**MF115744**	**MF115753**
		**MF115721**			
DE	Deserta Grande	**MF115722**	**MF115742**	**MF115751**	**MF115760**
MA1	Madeira: Canico Baixo	**MF115723**	**MF115735**	**MF115745**	**MF115754**
		**MF115724**	**MF115736**	**MF115746**	**MF115755**
MA2	Madeira: São Gonçalo	**MF115725**	**MF115737**	**MF115747**	**MF115756**
		**MF115726**	**MF115738**	**MF115748**	**MF115757**
		**MF115727**	**MF115739**		
		**MF115728**			
MA3	Madeira: Santa Cruz	**MF115729**	**MF115740**	**MF115749**	**MF115758**
		**MF115730**	**MF115741**	**MF115750**	**MF115759**
		**MF115731**			
		**MF115732**			
		Scrophularia arguta			
FU1	Canary Islands: Fuerteventura, Tetir	KU945636	KU926629	KU945695	KU945756
		KU945637	KU926630	KU945696	KU945757
FU2	Canary Islands: Fuerteventura, Tiscamanita	KU945638	KU926631	KU945697	KU945758
		KU945639	KU926632	KU945698	KU945759
GC	Canary Islands: Gran Canaria, La Isleta	KU945690	KU926684	KU945750	KU945811
		KU945691	KU926685	KU945751	KU945812
GO	Canary Islands: La Gomera, Barranco de Guarimiar	KU945694	KU926686	KU945752	KU945813
IB1	Spain: Cáceres, Santiago de Alcántara	KU945640	KU926635	KU945700	KU945762
		KU945641	KU926636	KU945701	KU945763
IB2	Spain: Almería, Pulpí	KU945642	KU926637	KU945702	KU945764
		KU945643	KU926638	KU945703	KU945765
LA1	Canary Islands: Lanzarote, Jameos del Agua	KU945648	KU926643	KU945708	KU945770
		KU945649	KU926644	KU945709	KU945771
LA2	Canary Islands: Lanzarote, Tinajo	KU945654	KU926649	KU945714	KU945776
		KU945655	KU926650	KU945715	KU945777
MO1	Morocco: Safi Cape	KU945658	KU926653	KU945718	KU945780
		KU945659	KU926654	KU945719	KU945781
MO2	Morocco: Zegangane	KU945664	KU926659	KU945724	KU945786
		KU945665	KU926660	KU945725	KU945787
MO3	Morocco: Hassi-Berkane	KU945666	KU926661	KU945726	KU945788
		KU945667	KU926662	KU945727	KU945789
MO4	Morocco: Had-Rouadi	KU945668	KU926663	KU945728	KU945790
		KU945669	KU926664	KU945729	KU945791
MO5	Morocco: Beni-Sidel	KU945670	KU926665	KU945730	KU945792
		KU945671	KU926666	KU945731	KU945793
MO6	Morocco: Sidi-Bou-Othmane	KU945672	KU926667	KU945732	KU945794
		KU945673	KU926668	KU945733	KU945795
MO7	Morocco: Oued El-Abid Gorges	KU945674	KU926669	KU945734	KU945796
		KU945675	KU926670	KU945735	KU945797
MO8	Morocco: Ouzaghar	KU945676	KU926671	KU945736	KU945798
		KU945677	KU926672	KU945737	KU945799
MO9	Morocco: Oued Assaka	KU945678	KU926673	KU945738	KU945800
		KU945679	KU926674	KU945739	KU945801
MO10	Morocco: Beddouza	KU945660	KU926655	KU945720	KU945782
		KU945661	KU926656	KU945721	KU945783
PA	Canary Islands: La Palma, Santa Cruz	KU945692	KU926687	KU945753	KU945814
		KU945693	KU926688	KU945754	KU945815

SA1	Saudi Arabia: Jabal Hada	KU945680	KU926675	KU945741	KU945802
SA2	Saudi Arabia: Al-Baha	KU945681	KU926676	KU945742	KU945803
SO	Yemen: Socotra Island, Fiheri Park	KU945688	KU926683	KU945749	KU945810
SU	Sudan: Arkawit, Jebel Elsit	KU945682	KU926677	KU945743	KU945804
		KU945683	KU926678	KU945744	KU945805
TE1	Canary Islands: Tenerife, Güimar	KU945684	KU926679	KU945745	KU945806
		KU945685	KU926680	KU945745	KU945807
TE2	Canary Islands: Tenerife, Pal-Mar	KU945686	KU926681	KU945747	KU945808
		KU945687	KU926682	KU945748	KU945809
Outgroup					
S. megalantha		KU945689	KC692563	KU945740	KU945755

Sequences in bold were generated in this study. Five additional sequences of each marker region obtained for populations PA, TE1 and TE2 of *S. arguta* were identical to sequences obtained in a previous study and were thus not submitted to GenBank
