# Supplementary material for: Peripatric speciation in an endemic Macaronesian plant after recent divergence from a widespread relative
Source: PLoS One. 2017 Jun 2;12(6):e0178459. doi: 10.1371/journal.pone.0178459 (PMC5456078; doi:10.1371/journal.pone.0178459)
Supplement: S2 Table — Taxa included in the ITS dataset for dating the origin and diversification of Scrophularia lowei, including GenBank accession numbers (GBN). Scrophularia lowei and S. arguta samples used in this analysis were the same as those given in S1 Table. (PDF) [file pone.0178459.s002.pdf]

S2 Table. Taxa included in the ITS dataset for dating the origin and diversification of *Scrophularia lowei*, including GenBank accession numbers (GBN). *Scrophularia lowei* and *S. arguta* samples used in this analysis were the same as those given in Table S1.

Taxa	GBN
Scrophularia atrata Pennell	HQ130081
Scrophularia auriculata L.	KC692527
Scrophularia buergeriana Miq.	HQ130070
Scrophularia bourgeana Lange in Willk. & Lange	KC692528
Scrophularia californica subsp. *floribunda* (Greene) R.J. Shaw	KR361737
Scrophularia calliantha Webb & Berthel.	KC692530
Scrophularia canina subsp. *bicolor* (Sm.) Greuter	KC692532
Scrophularia catariifolia Boiss. & Heldr.	KC692534
Scrophularia chrysantha Jaub. & Spach	KC692535
Scrophularia cinerascens Boiss.	KR361738
Scrophularia crassipedunculata Attar & Joharchi	KC692537
Scrophularia crassiuscula Grau	KC692538
Scrophularia deserti Delile	KR361739
Scrophularia desertorum (Munz) R.J. Shaw	HQ130087
Scrophularia duplicatoserrata Makino	HQ130076
Scrophularia eggersii Urb.	HQ130090
Scrophularia eriocalyx Emb. & Maire	KC692540
Scrophularia floribunda Boiss. & Balansa	KC692541
Scrophularia fontquerii Ortega Oliv. & Devesa	KC692542
Scrophularia frutescens L.	KR361740
Scrophularia glabrata Aiton	KR361741
Scrophularia grandiflora DC.	KC692545
Scrophularia grayana Maxim. ex Kom.	KC692546
Scrophularia herminii Hoffmanns. & Link	KR361742
Scrophularia heterophylla Willd.	KR361743
Scrophularia hirta Lowe	KC692550
Scrophularia hypericifolia Wydler	KC692551
Scrophularia ilwensis K. Koch	KR361745
Scrophularia jallui (Gattef. & Weiller) Ibn Tattou	KC692553
Scrophularia kakudensis Franch.	HQ130075
Scrophularia koraiensis Nakai	EU165340
Scrophularia kurdica subsp. *glabra* Grau	JF409905
Scrophularia laevigata var. *pubescens* Maire	KR361746
Scrophularia laevis Wooton & Standl.	HQ130077
Scrophularia lanceolata Pursh.	KC692555
Scrophularia laxiflora Lange	KC692556
Scrophularia lepidota Boiss.	KC692557
Scrophularia libanotica Boiss.	KC692558
Scrophularia lucida L.	KC692560
Scrophularia lunariifolia Boiss. & Balansa ex Boiss.	KC692558
Scrophularia lyrata Willd.	KR361747

Scrophularia macrantha Greene ex Stiefelh.	HQ130092
Scrophularia marilandica L.	HQ130085
Scrophularia megalantha Rech. f.	KC692563
Scrophularia micrantha Desv. ex Ham.	HQ130088
Scrophularia minutiflora Pennell	HQ130091
Scrophularia montana Wooton	HQ130082
Scrophularia multiflora Pennell	HQ130078
Scrophularia musashiensis Bonati	HQ130073
Scrophularia cf. *myriophylla* Boiss. & Heldr.	KC692565
Scrophularia ningpoensis Hemsl.	KC692566
Scrophularia olympica Boiss.	KC692568
Scrophularia orientalis L.	KC692569
Scrophularia oxyrrhyncha Coincy	KC692570
Scrophularia pauciflora Benth.	KC692571
Scrophularia peregrina L.	KU926689
Scrophularia peyronii Post	KC692573
Scrophularia pinardii Boiss.	KC692574
Scrophularia racemosa Lowe	KR361748
Scrophularia reuteri Daveau	KC692575
Scrophularia rosulata Stiefelh.	KC692577
Scrophularia sambucifolia L.	KC692579
Scrophularia scopolii Hoppe ex Pers.	KR361749
Scrophularia scorodonia L.	KC692582
Scrophularia serrata Rydb.	HQ130083
Scrophularia smithii Hornem.	KC692583
Scrophularia sublyrata Brot.	KC692584
Scrophularia tanacetifolia Willd.	KC692585
Scrophularia cf. *trichopoda* Boiss. & Balansa	KC692587
Scrophularia trifoliata L.	KC692588
Scrophularia umbrosa Dumort.	KR361751
Scrophularia valdesii Ortega Oliv. & Devesa	KC692590
Scrophularia viciosoi Ortega Oliv. & Devesa	KC692592
Scrophularia xylorrhiza Boiss. & Hausskn. ex Boiss.	KC692594
Scrophularia yoshimurae T. Yamaz	HQ130072
Outgroups	
Teedia lucida Rudolphi	AF375148
Verbascum nigrum L.	HQ130064
Verbascum virgatum Stokes	KC692522
