# Supplementary material for: Peripatric speciation in an endemic Macaronesian plant after recent divergence from a widespread relative
Source: PLoS One. 2017 Jun 2;12(6):e0178459. doi: 10.1371/journal.pone.0178459 (PMC5456078; doi:10.1371/journal.pone.0178459)
Supplement: S3 Table — Characteristics of DNA sequence datasets and number of unambiguous indels used in the analysis of Scrophularia lowei and S. arguta. (PDF) [file pone.0178459.s003.pdf]

S3 Table. Characteristics of DNA sequence datasets and number of unambiguous indels used in the analysis of *Scrophularia lowei* and *S. arguta*.

	Nuclear DNA	Chloroplast DNA
Alignment length (bp)	963	1522
Sequence length excluding gaps*	954–956 (950)	1296–1441 (1483)
Pairwise % identity	99.1 %	92.4 %
Variable characters	55	66
Unambiguous complex indels	-	9

* Data from *S. arguta* and *S. lowei* sequences; numbers in parentheses are from the outgroup
