# Supplementary material for: Peripatric speciation in an endemic Macaronesian plant after recent divergence from a widespread relative
Source: PLoS One. 2017 Jun 2;12(6):e0178459. doi: 10.1371/journal.pone.0178459 (PMC5456078; doi:10.1371/journal.pone.0178459)
Supplement: S4 Table — (PDF) [file pone.0178459.s004.pdf]

**S4 Table. Chloroplast DNA sequence variation in populations with haplotype G.**

| Population | Matrix position | | | |
|---|---|---|---|---|
| | 9 | 154–166 | 319 | 346 |
| *Scrophularia lowei* | | | | |
| AZ | T | 10 T | A | - |
| DE | T | 9 T | - | - |
| MA1 | T | 13 T | A | - |
| MA2 | T | 10 T | A | - |
| MA3 | T | 13 T | A | - |
| *Scrophularia arguta* | | | | |
| GO | T | 12 T | A | T |
| PA | T / A* | 10 T | A | T / A* |
| TE1 | T | 10 T | A | A |
| TE2 | T | 10 T | A | T |

* T in six individuals and A in one individual
