# Supplementary material for: Peripatric speciation in an endemic Macaronesian plant after recent divergence from a widespread relative
Source: PLoS One. 2017 Jun 2;12(6):e0178459. doi: 10.1371/journal.pone.0178459 (PMC5456078; doi:10.1371/journal.pone.0178459)
Supplement: S5 Table — Bayes factor (BF) support for significant connections (BF > 3) between geographical areas based on BSSVS analysis of chloroplast DNA in Scrophularia lowei and S. arguta using symmetrical and asymmetrical models. Only connections including S. lowei populations are indicated. (PDF) [file pone.0178459.s005.pdf]

S5 Table. Bayes factor (BF) support for significant connections (BF > 3) between geographical areas based on BSSVS analysis of chloroplast DNA in *Scrophularia lowei* and *S. arguta* using symmetrical and asymmetrical models. Only connections including *S. lowei* populations are indicated.

Geographical area connection	BF
Symmetrical model	
Sudan / Madeira	20.07
Arabia / Azores	14.65
NW Africa / Azores	13.93
Gran Canaria / Madeira	3.94
Lanzarote / Azores	3.51
Fuerteventura / Azores	3.21
Asymmetrical model	
Azores / Arabia	17.78
Sudan / Madeira	13.67
NW Africa / Azores	12.26
Azores / Fuerteventura	8.97
Madeira / Sudan	8.45
Azores / NW Africa	8.28
Azores / Lanzarote	7.96
Gran Canaria / Madeira	6.82
Arabia / Azores	3.46
