# Supplementary material for: Peripatric speciation in an endemic Macaronesian plant after recent divergence from a widespread relative
Source: PLoS One. 2017 Jun 2;12(6):e0178459. doi: 10.1371/journal.pone.0178459 (PMC5456078; doi:10.1371/journal.pone.0178459)
Supplement: S6 Table — (PDF) [file pone.0178459.s006.pdf]

S6 Table. Assignment of populations to cluster and changes in logarithm of marginal likelihood (log(ML)) when populations were transferred to the no selected cluster.

Population	Cluster	log (ML)
	Scrophularia. lowei	
AZ	1	-192.5112
MA1	1	-146.6127
MA2	1	-233.9670
MA3	1	-231.0106
DE	1	-78.2153
	Scrophularia. arguta	
FU1	2	-306.1345
FU2	2	-293.7921
GC	2	-148.8186
GO	1	-128.3146
IB1	2	-130.4218
IB2	2	-175.6867
LA1	2	-305.3650
LA2	2	-302.8665
MO1	2	-137.5522
MO2	2	-145.1212
MO3	2	-144.4816
MO4	2	-137.3188
MO5	2	-146.3050
MO6	2	-177.3061
MO7	2	-149.9517
MO8	2	-187.5543
MO9	2	-172.0268
MO10	2	-141.3379
PA	1	-82.7287
SA1	2	-135.9762
SA2	2	-135.9762
SO	2	-124.6521
SU	2	-180.4574
TE1	1	-118.5226
TE2	1	-108.6324
