# Supplementary figures and images for: Peripatric speciation in an endemic Macaronesian plant after recent divergence from a widespread relative
Source: PLoS One. 2017 Jun 2;12(6):e0178459. doi: 10.1371/journal.pone.0178459 (PMC5456078; doi:10.1371/journal.pone.0178459)

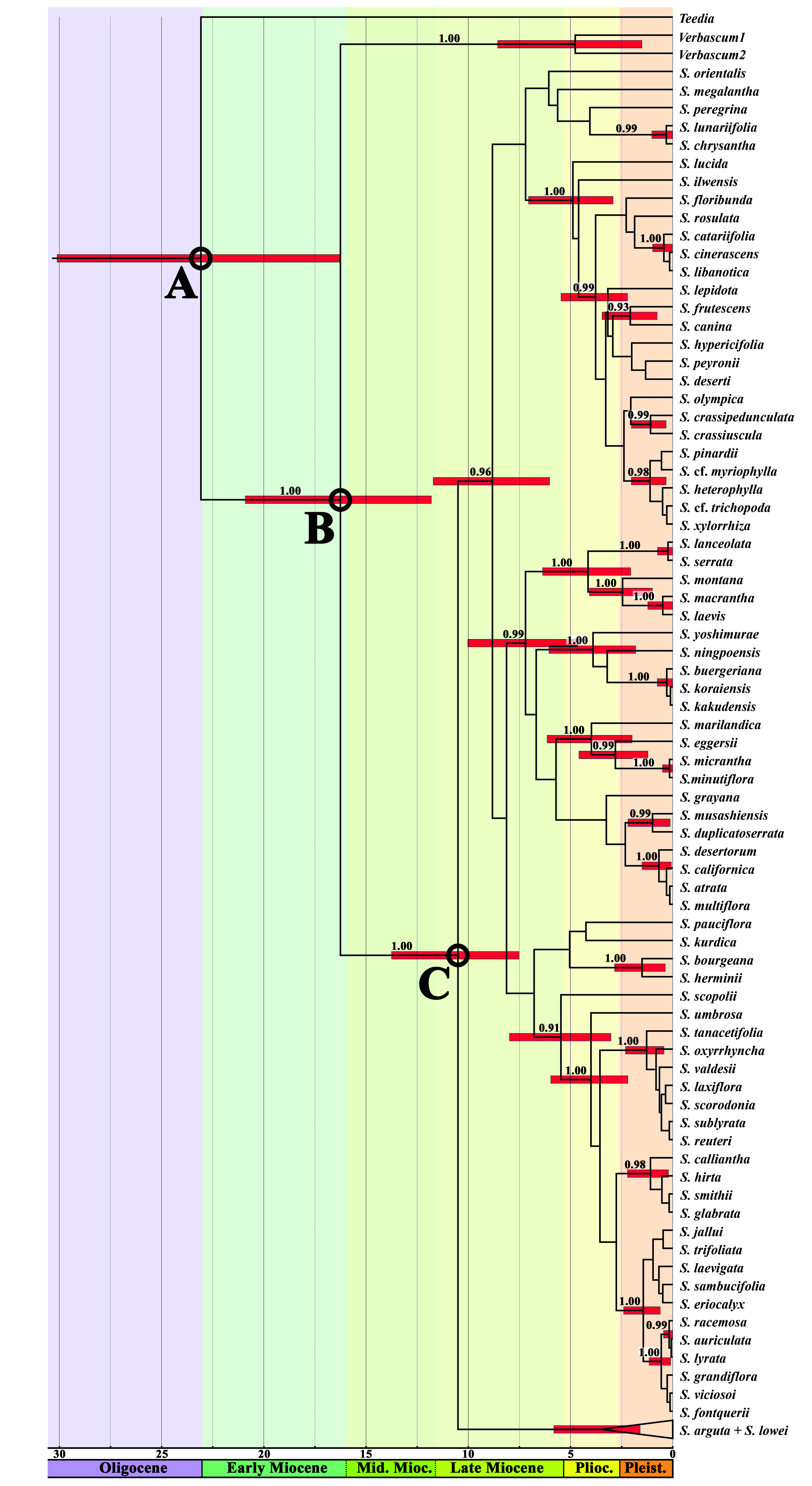

Supplement: S1 Fig — BEAST chronogram of Scrophularia based on ITS sequence variation. Posterior probabilities of clades are indicated above branches (only PP ≥ 0.90). The 95% posterior density distribution of node ages is shown in the node bars (only branches with a PP ≥ 0.90). The scale is in million years. Arrows indicate calibration points used in the analysis (A, 26.77 ± 4.27 Ma; B, 15.92 ± 3.29 Ma; C, 10.20 ± 2.36 Ma). (TIF) [file pone.0178459.s007.tif]

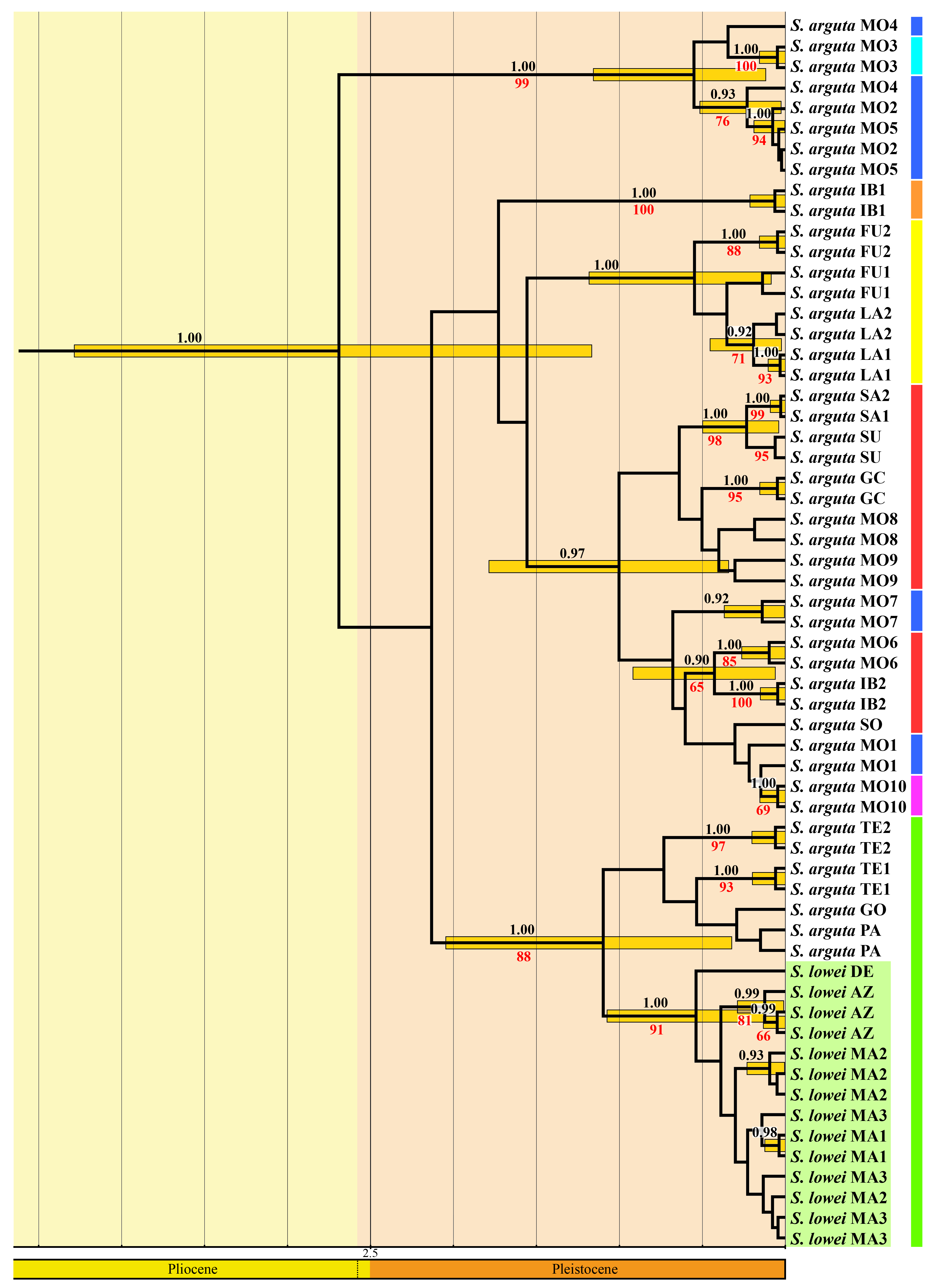

Supplement: S2 Fig — Black and red numbers above and below branches are posterior probability (PP) and maximum likelihood (ML) bootstrap (BS) values, respectively. Only values corresponding to a PP ≥ 0.90 and a ML BS ≥ 65 are shown. The light green background indicates S. lowei populations. Colours on the right correspond to the main haplotype indicated as in Fig 5. (TIF) [file pone.0178459.s008.tif]

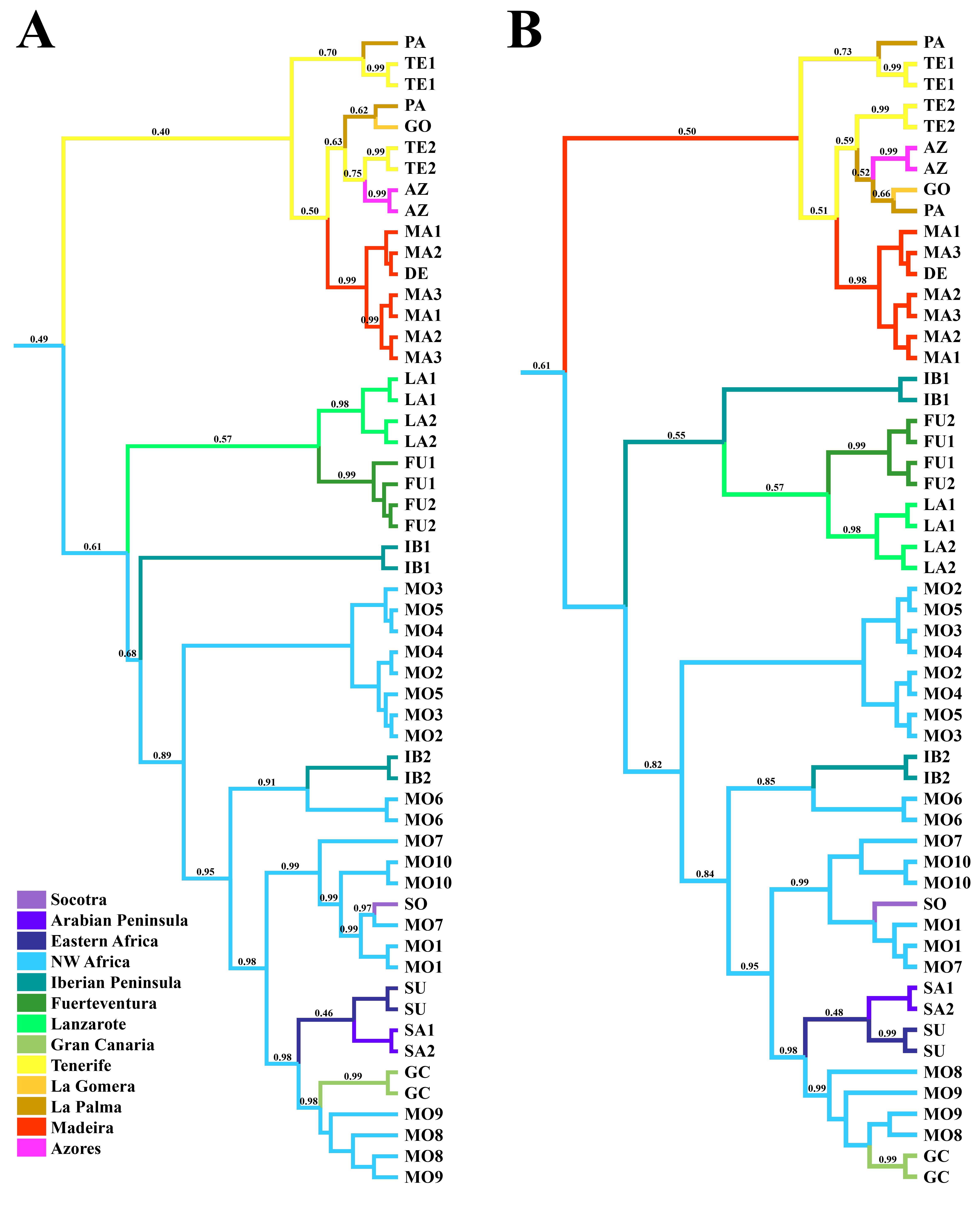

Supplement: S3 Fig — Maximum clade credibility tree generated by BSSVS analysis of cpDNA in Scrophularia lowei and S. arguta considering symmetrical (A) and asymmetrical (B) models. Branches are colored according to highest probability inferred ancestral geographical range. Highest probability of geographical range is indicated above branches (only values < 1.00). (TIF) [file pone.0178459.s009.tif]
